# KAT6B::KANSL1 fusion-positive uterine mesenchymal neoplasm with hybrid endometrial stromal and smooth muscle features: a case report

**DOI:** 10.3389/fmed.2026.1859849

**Published:** 2026-10-13

**Authors:** Qin Li, Meijuan Wu

**Affiliations:** 1 The Second Clinical Medical College of Zhejiang Chinese Medical University, Hangzhou, China; 2 Zhejiang Cancer Hospital, Hangzhou, China

**Keywords:** endometrial stromal sarcoma, KAT6B::KANSL1 fusion, molecular diagnostics, smooth muscle neoplasm, uterine sarcoma

## Abstract

KAT6B/A::KANSL1 fusion-positive uterine mesenchymal neoplasms are rare and may show overlapping endometrial stromal and smooth muscle features. We report a 47-year-old woman with regular menses, no dysmenorrhea, and a 7-year history of a presumed uterine fibroid. She underwent laparoscopic myomectomy at an outside hospital in November 2024. The fragmented specimen was interpreted as an endometrial stromal tumor with smooth muscle differentiation, but the tumor–myometrium interface could not be assessed, and an endometrial stromal nodule could not be distinguished from low-grade endometrial stromal sarcoma. Subsequent pelvic magnetic resonance imaging showed postoperative changes, possible residual leiomyomas, and heterogeneous enhancement at the uterine fundus. Pelvic computed tomography showed heterogeneous uterine enhancement without definite extrauterine disease. Because the original specimen was fragmented and a potentially malignant uterine mesenchymal neoplasm could not be excluded, completion hysterectomy with bilateral salpingo-oophorectomy was performed. The hysterectomy specimen contained multiple residual intramyometrial nodules with marked regional heterogeneity. One component showed fascicular smooth muscle morphology and diffuse desmin and h-caldesmon expression, whereas other nodules showed tongue-like myometrial infiltration, focal intravascular growth, spiral arteriole-like vessels, and diffuse ER, PR, CD10, and SMA expression. Whole-transcriptome sequencing identified an in-frame KAT6B::KANSL1 fusion involving KAT6B exon 3 and KANSL1 exon 11. Because the tumor expressed ER and PR, postoperative letrozole was administered for 30 days, after which surveillance was selected following review of the final pathologic and molecular findings. At 8 months after completion surgery, no clinical or radiologic recurrence was detected; this limited follow-up does not permit conclusions regarding long-term biological behavior or endocrine treatment benefit.

## 1 Introduction

Uterine mesenchymal neoplasms are a heterogeneous group of tumors showing smooth muscle, endometrial stromal, or less clearly defined lines of differentiation. Their classification has become increasingly complex as new morphologic subtypes and recurrent molecular alterations have been identified (1–3). Histology and immunohistochemistry remain central to diagnosis, but considerable overlap occurs among uterine spindle cell neoplasms, particularly when cytologic atypia and proliferative activity are limited (4, 5). In such cases, an integrated approach that includes molecular testing may be required for reliable classification (3, 6, 7).

Uterine tumors harboring KAT6B/A::KANSL1 fusions illustrate this diagnostic problem. They are not currently listed as a separate entity in the WHO Classification of Female Genital Tumours and are best regarded, at present, as an emerging molecularly defined group (3, 8–10). Early reports described the same rearrangement in tumors interpreted as leiomyoma or leiomyosarcoma (11, 12). Subsequent series showed a more consistent pattern of bland spindle or ovoid cells, low proliferative activity, fibrous or fibromyxoid stroma, and variable endometrial stromal and smooth muscle differentiation (8–10, 13). Gene-expression and DNA methylation studies further suggest that many of these tumors are molecularly distinct from conventional endometrial stromal and smooth muscle neoplasms (9, 10). Some authors have therefore proposed that they represent a distinct type of uterine sarcoma, although their formal taxonomic position and full spectrum of biological behavior remain unsettled (8–10, 13). The main diagnostic difficulty is their resemblance to both low-grade endometrial stromal sarcoma and smooth muscle tumors. Features such as permeative myometrial growth, lymphovascular involvement, spiral arteriole-like vessels, and hormone-receptor expression may suggest low-grade endometrial stromal sarcoma (8, 10, 14, 15). Other areas may show fascicular architecture and smooth muscle marker expression, closely mimicking cellular leiomyoma or another smooth muscle neoplasm (8–10, 13). This distinction has practical consequences. Although many reported tumors appear histologically low grade, recurrence and aggressive clinical behavior have occurred in a subset (8–10, 13). An accurate diagnosis may therefore affect the adequacy of limited local excision, the choice of definitive surgery, and the need for prolonged surveillance. The role of endocrine therapy remains uncertain despite frequent estrogen and progesterone receptor expression. More broadly, expert review and molecular analysis can substantially alter the classification of rare uterine mesenchymal tumors (16).

Here, we report a KAT6B::KANSL1 fusion-positive uterine mesenchymal neoplasm with marked regional variation in morphology and immunophenotype. Some areas closely resembled a smooth muscle tumor, whereas others showed features overlapping with low-grade endometrial stromal sarcoma. By presenting the clinicopathologic, immunohistochemical, and molecular findings, we aim to highlight the diagnostic pitfalls of this emerging group and the value of fusion testing in uterine mesenchymal tumors that cannot be confidently classified by morphology and immunohistochemistry alone.

## 2 Case presentation

A 47-year-old woman was referred to our institution in May 2025 for further evaluation of a uterine mesenchymal neoplasm after previous surgery at an outside hospital. She had been aware of a uterine fibroid for more than 7 years. No specific symptoms were documented at her initial presentation to the outside hospital. Her menstrual cycles were regular, without dysmenorrhea, and she had one son. Pelvic ultrasonography performed on November 12, 2024, showed an intrauterine device, a uterine fibroid, and bilateral cystic ovarian lesions considered non-neoplastic. On November 18, 2024, she underwent laparoscopic myomectomy, removal of the intrauterine device, excision of a right ovarian lesion, and excision of a right mesosalpinx lesion. Histologic examination showed a simple cyst of the right ovary, a right mesosalpinx cyst, and a uterine endometrial stromal tumor with smooth muscle differentiation. Because the uterine specimen was fragmented, the tumor–myometrium interface could not be assessed, and an endometrial stromal nodule could not be reliably distinguished from low-grade endometrial stromal sarcoma. An adenomatoid tumor was also identified. A pathological consultation at another institution on March 17, 2025, favored a low-grade spindle cell mesenchymal neoplasm with partial myoid differentiation. The tumor cells were positive for SMA, desmin, and ER, showed focal h-caldesmon expression, and were negative for calponin, HMB45, and myogenin; the Ki-67 labeling index was approximately 1%. Apart from the long-standing uterine fibroid and prior intrauterine device placement, she had no other relevant gynecologic history. She had no significant medical history, no other history of extrauterine surgery, and no family history of gynecologic malignancy or other cancers.

Pelvic magnetic resonance imaging performed on May 10, 2025, showed postoperative changes after myomectomy, possible residual leiomyomas, and heterogeneous enhancement at the uterine fundus. Multiple cervical cysts, a possible perineal cyst, and a small amount of pelvic fluid were also noted. Subsequent unenhanced and contrast-enhanced pelvic computed tomography at our institution showed heterogeneous uterine enhancement, probable physiologic changes in both adnexal regions, and a small amount of pelvic fluid, without definite radiologic evidence of extrauterine tumor. Review of the available pathological material at our institution supported a low-grade spindle cell mesenchymal neoplasm, but definitive subclassification and assessment of biological behavior remained difficult because the original specimen was fragmented. Given the concern for a potentially malignant uterine mesenchymal neoplasm and the inability to exclude low-grade endometrial stromal sarcoma, definitive surgery was recommended after multidisciplinary review. On May 20, 2025, the patient underwent transabdominal extended total hysterectomy, bilateral salpingo-oophorectomy, and adhesiolysis. On gross examination, multiple gray-white nodules were identified within the myometrium. The nodules were irregular in shape and firm in consistency. Some were relatively well circumscribed (Figures 1A,B), whereas others had indistinct borders (Figure 1C). The largest nodule measured 1.5 cm in greatest dimension. No gross abnormality was identified in the endometrium, cervix, bilateral fallopian tubes, or ovaries.

**Figure 1:**
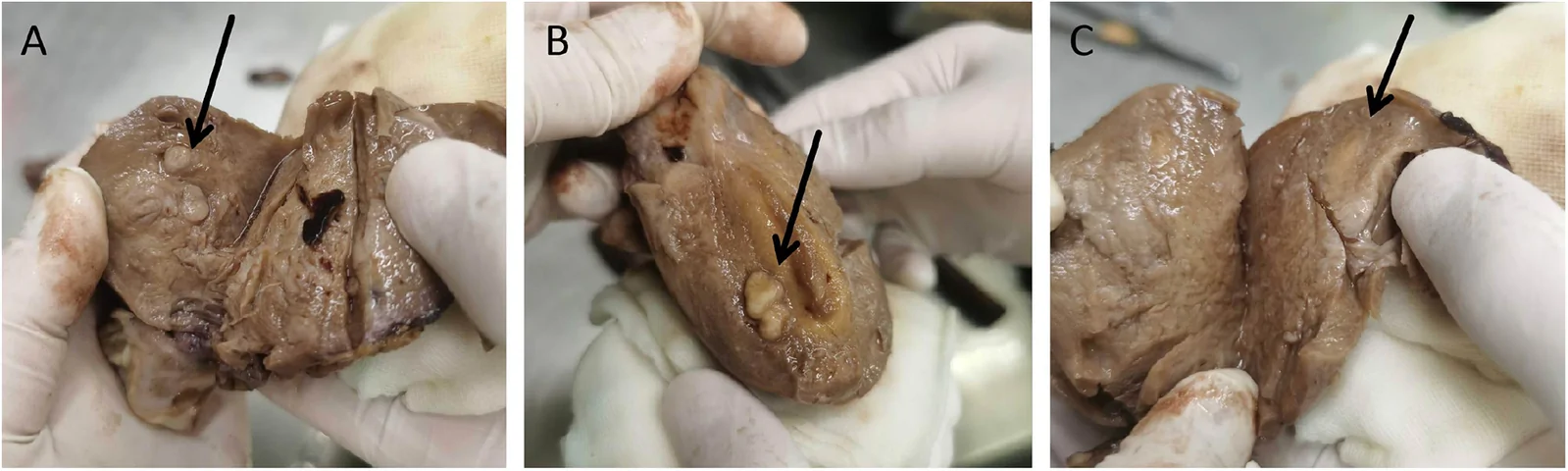
Gross features of the uterine lesion. **(A–C)** Cut surfaces of the hysterectomy specimen show multiple gray-white, firm myometrial nodules (arrows). The nodules are irregular in shape and exhibit variable borders, ranging from relatively well circumscribed to poorly defined.

Microscopically, multiple nodular lesions were identified within the myometrium, showing conspicuous morphologic heterogeneity on low-power examination (Figure 2). One lesion on the left side was relatively well demarcated and regular in contour. It was composed of spindle cells arranged in intersecting fascicles. The tumor cells had eosinophilic cytoplasm and blunt-ended, cigar-shaped nuclei with a relatively low nuclear-to-cytoplasmic ratio, closely resembling a smooth muscle neoplasm (Figure 3A). In contrast, the right-sided nodules were poorly circumscribed and showed tongue-like infiltration into the surrounding myometrium and focal intravascular growth (Figure 3B). Compared with the left-sided lesion, these nodules were more cellular and were composed of tumor cells arranged in a whorled pattern, with short spindle nuclei and eosinophilic to clear cytoplasm (Figure 3C). The intervening stroma showed focal myxoid change (Figure 3D) and collagen deposition (Figure 3E). In addition, numerous vascular structures were present, including abundant spiral arteriole-like vessels and conspicuous thick-walled vessels. Short spindle cells were concentrically arranged around many of these vascular channels, producing a characteristic swirling appearance (Figure 3F). Cytologic atypia remained minimal, and no conspicuous mitotic activity was identified. Immunohistochemically, the left-sided lesion showed weak ER staining and diffuse strong expression of PR, SMA, desmin, and h-caldesmon. CD10, CD34, Melan-A, and Cyclin D1 were negative in this component. The Ki-67 labeling index was low, at approximately 5% (Figure 4). In contrast, the right-sided nodules showed diffuse strong positivity for ER, PR, CD10, and SMA, whereas desmin, h-caldesmon, CD34, Melan-A, and Cyclin D1 were negative. The Ki-67 labeling index in these areas was likewise approximately 5% (Figure 5). Overall, the immunophenotype of the left-sided lesion supported smooth muscle differentiation, whereas the right-sided nodules showed a hybrid immunophenotype characterized by diffuse ER, PR, and CD10 expression together with retained SMA positivity. Given the overlapping morphologic and immunophenotypic features of low-grade endometrial stromal sarcoma and smooth muscle tumor, molecular testing was undertaken to resolve the differential diagnosis.

**Figure 2:**
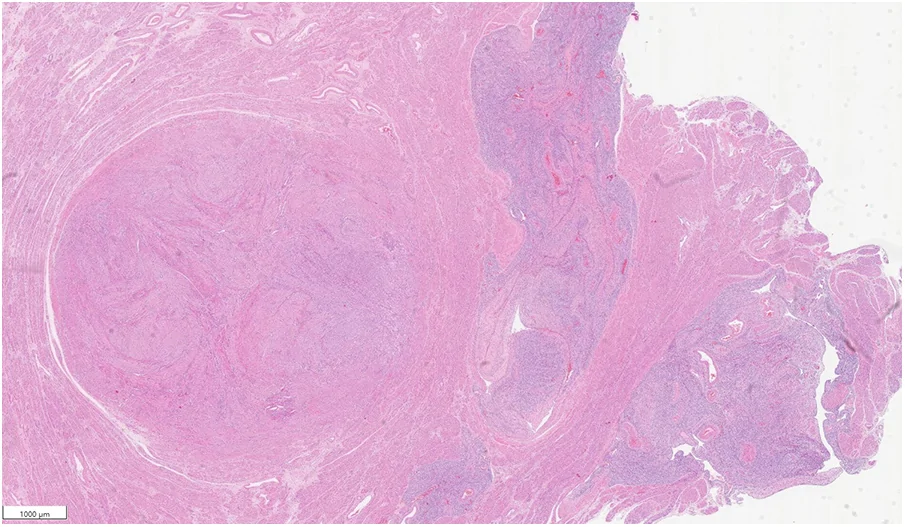
Low-power histologic features of the uterine lesion. Hematoxylin and eosin staining demonstrates multiple myometrial nodules with distinct architectural patterns. A relatively well-demarcated fascicular lesion is present on the left, whereas the lesions on the right are less circumscribed and show a more irregular growth pattern. Scale ba*r* = 1,000 μm.

**Figure 3:**
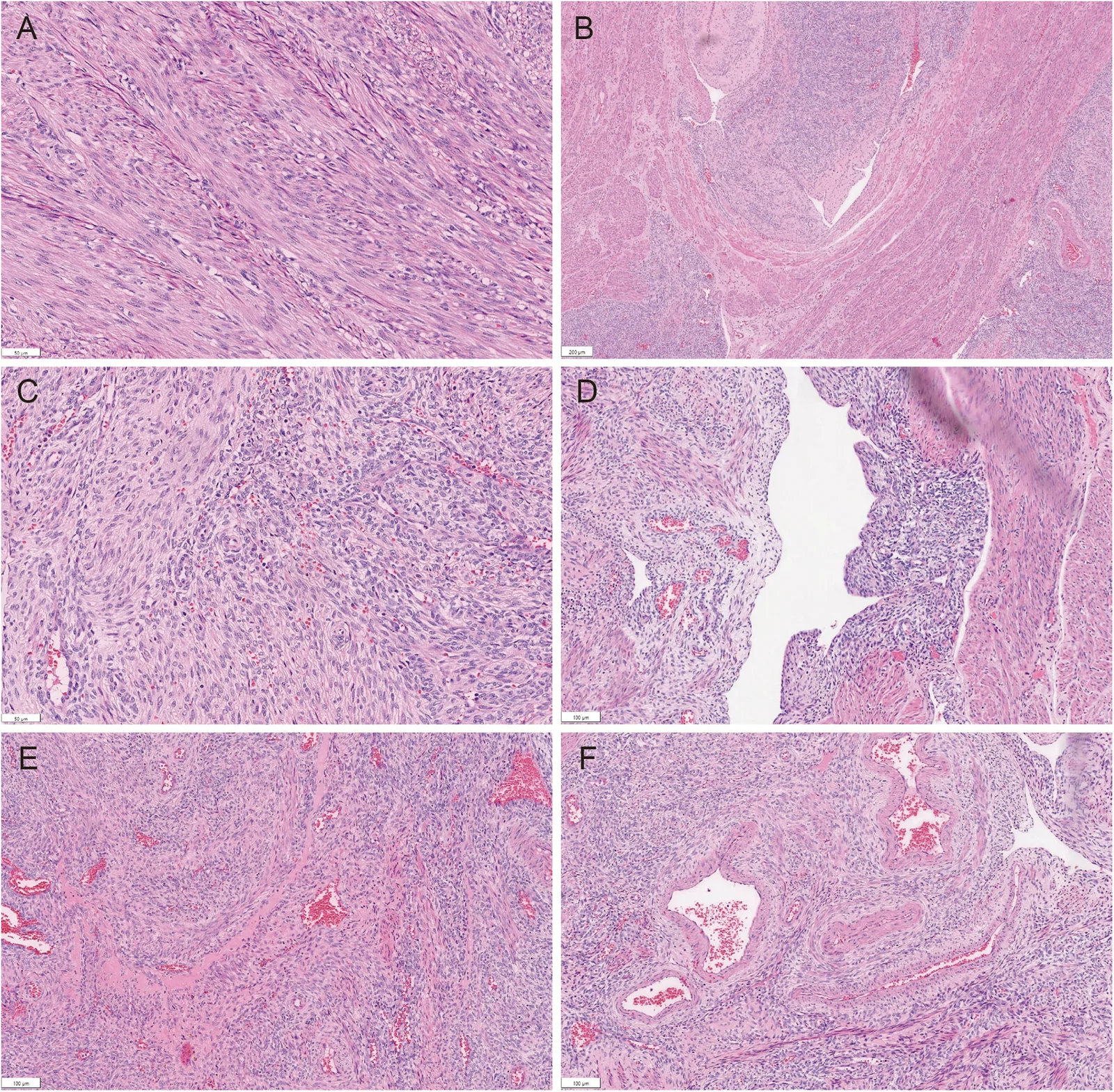
Microscopic findings of the uterine tumor (hematoxylin and eosin staining). **(A)** The left-sided lesion is composed of spindle cells with blunt-ended nuclei and a low nuclear-to-cytoplasmic ratio (×200). **(B)** The right-sided lesion shows tongue-like infiltration into the adjacent myometrium and intravascular growth (×40). **(C)** Tumor cells in the right-sided lesion are arranged in a whorled pattern, with short spindle nuclei and eosinophilic to clear cytoplasm (×200). **(D)** Focal myxoid stromal change is noted (×100). **(E)** Stromal collagen deposition is present (×100). **(F)** Numerous vascular structures, including spiral arteriole-like vessels and thick-walled vessels, are seen, with short spindle cells concentrically arranged around the vessels in a whorled pattern (×100).

**Figure 4:**
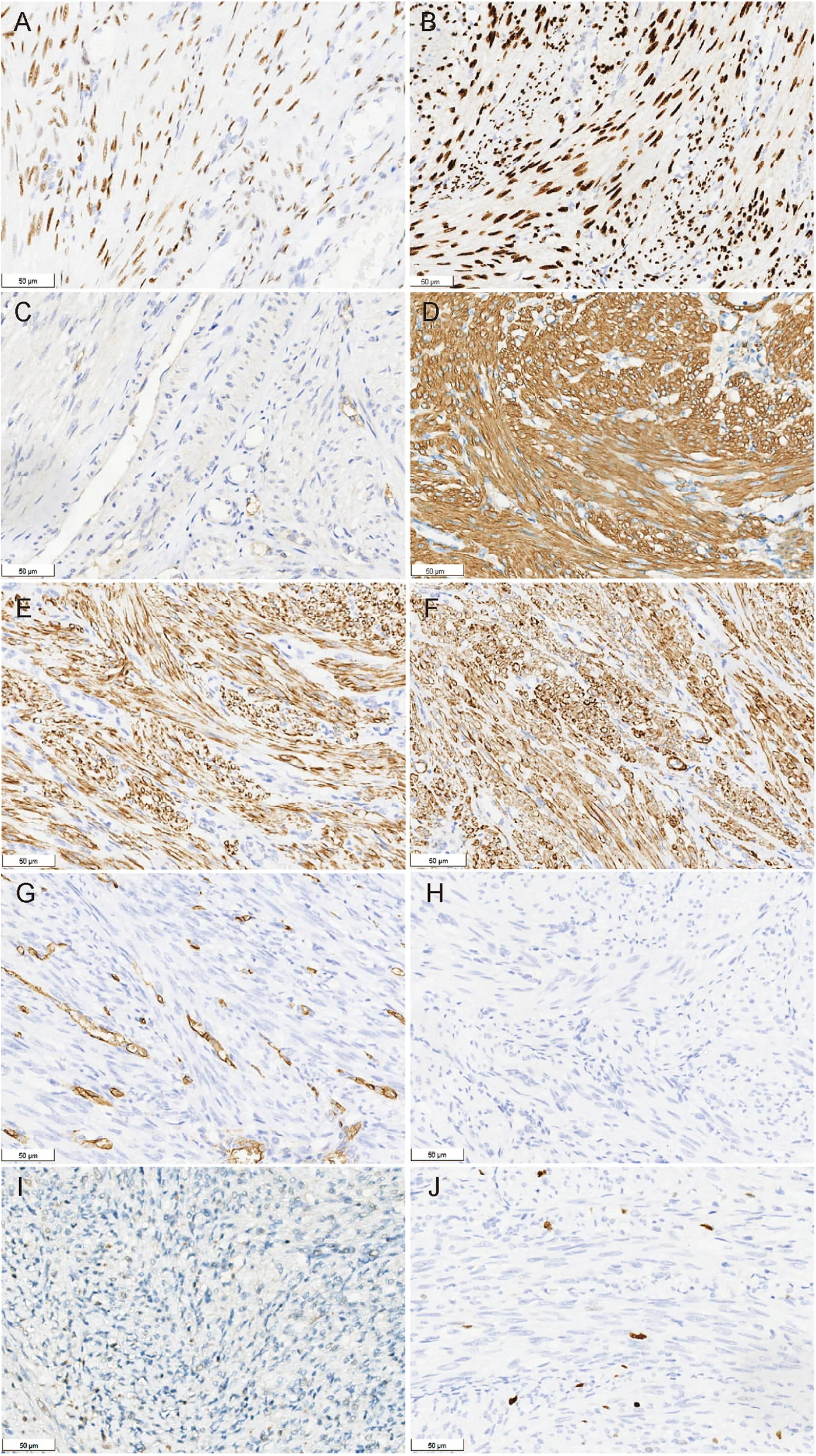
Immunohistochemical profile of the left-sided lesion. The tumor shows weak nuclear staining for ER **(A)**, diffuse strong nuclear staining for PR **(B)**, negative staining for CD10 **(C)**, diffuse strong positivity for SMA **(D)**, desmin **(E)**, and h-caldesmon **(F)**, negative staining for CD34 in tumor cells with positivity confined to vascular structures **(G)**, negative staining for Melan-A **(H)** and Cyclin D1 **(I)**, and a low Ki-67 labeling index **(J)** Scale ba*r* = 50 μm.

**Figure 5:**
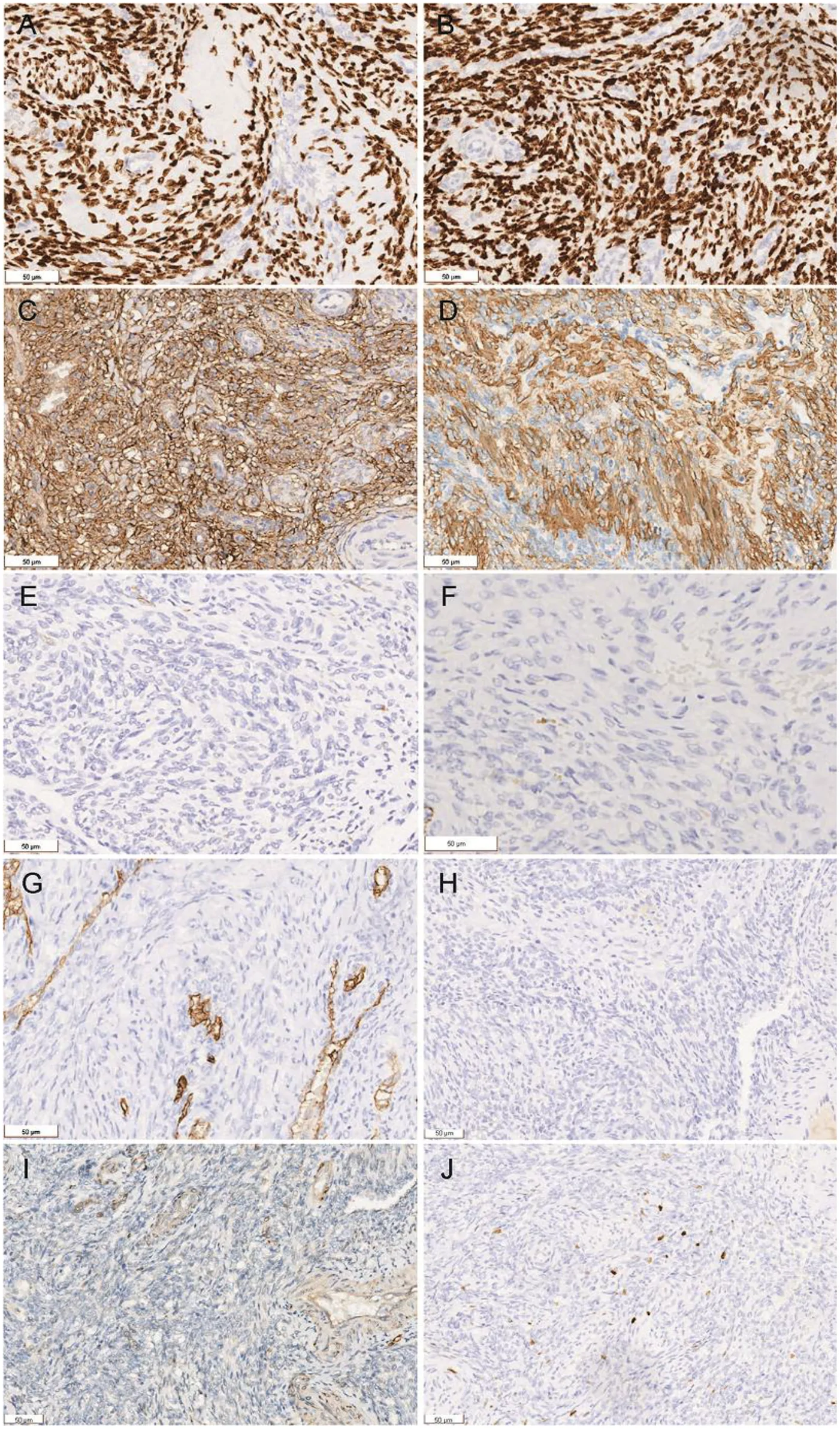
Immunohistochemical profile of the right-sided lesion. The tumor shows diffuse strong nuclear staining for ER **(A)** and PR **(B)**, diffuse positivity for CD10 **(C)** and SMA **(D)**, negative staining for desmin **(E)** and h-caldesmon **(F)**, negative staining for CD34 in tumor cells with positivity confined to vascular structures **(G)**, negative staining for Melan-A **(H)** and Cyclin D1 **(I)**, and a low Ki-67 labeling index **(J)**. Scale ba*r* = 50 μm.

For molecular analysis, whole-transcriptome sequencing was performed. Libraries were prepared using a human whole-transcriptome assay kit, and sequencing was carried out on an Illumina NextSeq CN550 platform. Variant interpretation and reporting followed the recommendations of the Association for Molecular Pathology, the American Society of Clinical Oncology, and the College of American Pathologists, and nomenclature was standardized according to Human Genome Variation Society criteria. Sequencing identified an in-frame KAT6B::KANSL1 fusion transcript involving exon 3 of KAT6B (10q22.31) and exon 11 of KANSL1 (17q21.31). Taken together with the histologic and immunohistochemical findings, these results supported the diagnosis of a KAT6B::KANSL1 fusion-positive uterine mesenchymal neoplasm. The postoperative recovery was uneventful. During 8 months of follow-up after completion surgery, surveillance was performed using serial pelvic ultrasonography and pelvic computed tomography; when CT was obtained, both unenhanced and contrast-enhanced images were acquired. Follow-up examinations showed mild thickening of the vaginal stump and a small amount of pelvic fluid, but no abnormal pelvic mass or radiologic evidence of recurrence. Because the tumor showed ER and PR expression, postoperative letrozole was initiated as adjuvant endocrine therapy at a dose of 2.5 mg once daily. After review of the final histopathologic and molecular findings, the treating team considered the risk of recurrence to be low. In view of the limited expected benefit and the potential adverse effects of continued treatment, letrozole was discontinued after 30 days. No further systemic therapy was administered, and surveillance with outpatient clinical assessment and pelvic imaging at approximately 6-month intervals was planned.

## 3 Discussion

Uterine mesenchymal neoplasms are increasingly being redefined by recurrent molecular alterations, particularly when a lesion cannot be assigned with confidence to a conventional category on morphologic and immunophenotypic grounds alone (2, 6). Within this evolving landscape, tumors harboring KAT6B/A::KANSL1 fusions have emerged as an uncommon molecularly defined group characterized by deceptively bland cytology, frequent overlap with low-grade endometrial stromal sarcoma (LG-ESS), and variable smooth muscle differentiation (8, 9). The present case is diagnostically significant because different areas showed divergent morphologic and immunophenotypic features, while molecular analysis identified a KAT6B::KANSL1 fusion. Its value therefore lies not only in its rarity, but also in showing why histology, immunohistochemistry, and fusion testing may all be needed when evaluating a difficult uterine spindle cell neoplasm.

Several aspects of the present tumor fall within the clinicopathologic spectrum reported for KAT6B/A::KANSL1-rearranged uterine mesenchymal neoplasms. Previous series have described bland spindle to ovoid cells, low proliferative activity, fibrous or fibromyxoid stroma, conspicuous arteriolar-type vasculature, and varying degrees of endometrial stromal and smooth muscle differentiation (8–10, 17). Our case showed many of these features, including a spindle cell proliferation with low-grade cytology, a whorled growth pattern in part of the tumor, focal myxoid and collagenous stromal change, and abundant spiral arteriole-like and thick-walled vessels. The presence of multiple intramyometrial nodules with different growth patterns is also consistent with the morphologic variability described in recent studies, in which some tumors were relatively well circumscribed whereas others showed infiltrative borders or vascular permeation (8, 15, 17). Although the histology was heterogeneous, it remained compatible with the expanding spectrum of this emerging molecular group.

The most striking feature of this case was its marked intratumoral heterogeneity. The left-sided lesion closely resembled a smooth muscle neoplasm, both morphologically and immunophenotypically, with intersecting fascicles of eosinophilic spindle cells and diffuse expression of desmin and h-caldesmon. In contrast, the right-sided nodules showed tongue-like myometrial infiltration, focal intravascular growth, whorled short spindle cells, conspicuous arteriole-like vasculature, and diffuse ER, PR, and CD10 expression with retained SMA positivity but loss of desmin and h-caldesmon. This regional divergence helps explain the diagnostic uncertainty generated by the original specimen and mirrors previous reports showing that KAT6B/A::KANSL1-rearranged tumors may occupy a histologic and immunophenotypic middle ground between endometrial stromal and smooth muscle neoplasms (8, 9, 17, 18). This hybrid pattern is an important clue that should prompt consideration of a KAT6B/A::KANSL1-rearranged uterine mesenchymal neoplasm in routine practice (10, 18).

The principal differential diagnoses were LG-ESS and a smooth muscle tumor. The right-sided nodules strongly suggested LG-ESS because of their tongue-like myometrial permeation, focal vascular invasion, spiral arteriole-like vessels, and diffuse ER, PR, and CD10 expression (14, 15). However, the separate component with overt smooth muscle morphology and diffuse desmin and h-caldesmon expression was not typical of conventional LG-ESS. Conversely, the left-sided lesion closely simulated a cellular smooth muscle tumor, but the accompanying right-sided component, with its stromal-type vascular pattern and diffuse CD10 positivity, was not compatible with a purely smooth muscle neoplasm (9, 10). High-grade ESS was less likely because the tumor lacked diffuse high-grade round cell morphology, marked nuclear atypia, brisk mitotic activity, tumor cell necrosis, and diffuse Cyclin D1 expression. Uterine PEComa was also unsupported because the tumor was negative for Melan-A (3, 4, 6, 14). When a uterine spindle cell tumor shows mixed stromal and myoid features that cannot be reconciled within a single established category, a fusion-defined uterine mesenchymal neoplasm should therefore be considered.

The initial laparoscopic myomectomy introduced additional diagnostic and management uncertainty. Because the specimen was fragmented, the tumor–myometrium interface and surgical margins could not be assessed reliably, and infiltrative or vascular growth may not have been adequately represented. The multiple intramyometrial nodules identified in the subsequent hysterectomy specimen also raise the possibility that multifocal residual disease remained after the initial procedure. Whether morcellation was used during the original operation could not be confirmed from the available records. Its possible effect on dissemination therefore cannot be determined, and no procedure-related spread was demonstrated during the current follow-up.

The evaluation of uterine mesenchymal tumors requires integration of clinical findings, imaging, histopathology, immunohistochemistry, and molecular testing. Cross-sectional imaging is useful at the initial assessment, particularly for defining tumor size, location, myometrial involvement, internal heterogeneity, hemorrhage or necrosis, and possible extrauterine extension. Computed tomography (CT) can help evaluate pelvic or distant disease, whereas magnetic resonance imaging (MRI) provides superior soft-tissue contrast and is often more informative for characterizing uterine masses and assessing local extent (19). Functional imaging, including single-photon emission computed tomography (SPECT) and positron emission tomography (PET), may provide complementary metabolic information and may be useful in selected cases for assessing disease extent or suspected recurrence (20, 21). However, imaging findings are not specific enough to reliably distinguish among the diverse histologic and molecular subtypes of uterine mesenchymal neoplasia. In diagnostically ambiguous tumors, as in the present case, imaging should therefore be interpreted together with morphology, immunophenotype, and, when indicated, fusion testing.

Molecular analysis was decisive in the present case. The detection of an in-frame KAT6B::KANSL1 fusion involving KAT6B exon 3 and KANSL1 exon 11 is noteworthy because this breakpoint has been recurrently documented and appears to be characteristic of this tumor group (12, 17, 22). Related rearrangements were initially identified in tumors diagnosed as leiomyoma or leiomyosarcoma. Subsequent clinicopathologic studies suggested that these lesions form a coherent molecularly defined group rather than representing an incidental alteration shared by unrelated neoplasms (8, 9). DNA methylation studies have further shown that KAT6B/A::KANSL1-positive uterine tumors form a distinct epigenetic cluster separate from conventional LG-ESS and other gynecologic mesenchymal neoplasms (10). The practical value of molecular testing is illustrated by the Rare Gynecologic Sarcoma Study, in which central review and molecular analysis led to reclassification of 29% of cases (16). RNA-based fusion testing should therefore be considered when a uterine spindle cell neoplasm shows hybrid endometrial stromal and smooth muscle features.

The clinical behavior of KAT6B/A::KANSL1-rearranged uterine mesenchymal neoplasms remains incompletely defined, and the limited published data suggest variable outcomes (8). Although many tumors show bland histologic features, recurrence, persistent disease, and disease-related death have been reported in a subset of patients (13). Methylation-based studies have suggested that tumors within a core low-grade group may follow a more favorable course than those with divergent morphologic or molecular findings, but the available evidence remains limited (10). At the latest follow-up, more than 8 months after completion surgery, the patient had no clinical or radiologic evidence of recurrence. This relatively short disease-free interval should not be interpreted as evidence of indolent biological behavior, successful treatment, or benefit from letrozole. The patient received letrozole for only 30 days, which is insufficient to assess treatment efficacy. Evidence supporting endocrine therapy, including aromatase inhibition, is derived mainly from conventional ER- and PR-positive LG-ESS (23, 24). There is currently no direct evidence that KAT6B::KANSL1-positive uterine mesenchymal neoplasms benefit from endocrine therapy, and the appropriate indications and treatment duration remain unknown. Long-term surveillance and multidisciplinary management are therefore warranted.

## 4 Conclusion

This case expands the clinicopathologic spectrum of KAT6B::KANSL1 fusion-positive uterine mesenchymal neoplasms. Its marked regional heterogeneity, with one component resembling a smooth muscle tumor and others overlapping with low-grade endometrial stromal sarcoma, explains why classification based on morphology and immunohistochemistry alone can be difficult. Whole-transcriptome sequencing identified an in-frame KAT6B::KANSL1 fusion and provided the decisive evidence for diagnosis. These findings support the use of RNA-based fusion testing in uterine spindle cell tumors with mixed stromal and myoid features. Because the formal classification and long-term behavior of this emerging molecular group remain incompletely defined, cautious terminology and continued clinical surveillance are warranted.

## Data Availability

The raw data supporting the conclusions of this article will be made available by the authors, without undue reservation.
